# Variability in the psychological impact of four waves of COVID-19: a time-series study of 60 000 text-based counseling sessions

**DOI:** 10.1017/S0033291722000587

**Published:** 2023-07

**Authors:** Christian S. Chan, Chi-Ting Yang, Yucan Xu, Lihong He, Paul S. F. Yip

**Affiliations:** 1 Department of Psychology, The University of Hong Kong, Hong Kong; 2 Center for Suicide Research and Prevention, HKU, Hong Kong

**Keywords:** Continuous traumatic stress, COVID-19, psychological distress, resilience, time-series

## Abstract

**Background:**

Continuous exposure to stressors can lead to vulnerability and, in some cases, resilience. This study examined the variation in its psychological impact across the first four waves of COVID-19 in Hong Kong.

**Methods:**

Transcripts from Open Up, an online text-based counseling service, between January 2019 and January 2021 were analyzed (*N* = 60 775). We identified COVID-19 mentioned sessions using keywords and further categorized them into those that also mentioned symptoms of common mental disorders (CMDs) and those that did not. Autoregressive integrated moving average models were used to analyze the associations between the severity of the outbreak and the mention of COVID-19 and CMDs.

**Results:**

Results revealed that the pandemic led to increased psychological distress. Compared to prior to its advent, more people sought help in the initial months of the outbreak. Furthermore, associations were found between the severity of the outbreak and the number of help-seeker mentioning the pandemic, as well as between the outbreak severity and the number of help-seekers disclosing psychological distress. However, these relationships were not uniform across the four waves of outbreaks; a dissociation between outbreak severity and help-seekers' concern was found in the fourth wave.

**Conclusion:**

As the pandemic waxes and wanes, people may become habituated to its psychological toll. This may be interpreted as a form of resilience. Instead of worsening with time, the psychological impact of COVID-19 may reduce with repeated exposure.

The mental health impact of the COVID-19 pandemic is alarming (Alzueta et al., 2021; Vindegaard & Benros, 2020). Emerging studies on youth, in particular, have documented the pandemic's impact on their mental health, daily life, and leisure (Courtney, Watson, Battaglia, Mulsant, & Szatmari, 2020; Elliott et al., 2021; Liang et al., 2020; Moore et al., 2020). Lockdowns and social distancing measures have substantially reduced social support and health-promoting activities, resulting in further rise of mental health issues (Elmer, Mepham, & Stadtfeld, 2020; Nearchou, Flinn, Niland, Subramaniam, & Hennessy, 2020; Niedzwiedz et al., 2021).

The psychological impact of disasters – both natural and human-caused – has been extensively examined (Beaglehole et al., 2018; Bonde et al., 2016; Labarda & Chan, 2018). However, prior to the COVID-19 pandemic, few studies have documented the effects of a potentially traumatizing event (PTE) that has affected as many people, for as long. Studies have found that repeated exposure to traumatic events creates a cumulative effect, rendering survivors more sensitive to subsequent traumatic events (*stress sensitization hypothesis*; e.g. Garfin, Holman, & Silver, 2015; Hammen, Henry, & Daley, 2000; McLaughlin, Conron, Koenen, & Gilman, 2010). On the other hand, researchers have maintained that, at least for some, repeated or protracted exposure to similar PTEs can strengthen resilience via habituation and coping (e.g. Bleich, Gelkopf, & Solomon, 2003). This study examined these two potentially competing hypotheses as the threat of COVID-19 persists.

## Sensitization hypothesis

Both laboratory studies and field research have shown that those exposed to stress may become more sensitive to subsequent events, and their responses would be heightened compared to those with fewer and less intensive prior exposure (Bandoli et al., 2017; Davies, Myers, Cummings, & Heindel, 1999; Smid et al., 2012). This sensitization hypothesis helps explain the variability in survivors' response to disasters and other PTEs (Dougall, Herberman, Delahanty, Inslicht, & Baum, 2000; Irish et al., 2008; Wilson et al., 2013). As the COVID-19 outbreak ebbs and flows, survivors might become more sensitive to the increase of risk. Early studies of the impact of COVID-19 suggest that psychological distress increases as the pandemic continues (Daly, Sutin, & Robinson, 2021; McGinty, Presskreischer, Han, & Barry, 2020).

## Habituation hypothesis

Continuous traumatic stress (CTS) refers to longer-term exposure to ongoing threats (Nuttman-Shwartz & Shoval-Zuckerman, 2016; Stevens, Eagle, Kaminer, & Higson-Smith, 2013). Several longitudinal studies demonstrated that CTS could lead to a reduction in the impact of PTE, resulting in resilience as opposed to worsening health and well-being (Bonanno et al., 2012; Ronen, Rahav, & Appel, 2011). In their longitudinal study in Israel, Stein, Levin, Gelkopf, Tangir, and Solomon (2018) found that increased exposure to armed conflicts was associated with *less* post-traumatic stress symptomology, not more. The authors interpreted this as the result of habituation; respondents became less distressed over time, and the symptoms presented earlier on were in fact adaptive responses and not traumatic reactions.

As the threat of the pandemic protracts, would those under continuous threat of exposure to the virus become psychologically inoculated to its associated stress? This study sought to examine the changes in the psychological reaction to the threat of the pandemic, especially its relationship with the ebbs and flows of outbreaks. This is particularly relevant, as many countries experience multiple waves of infections.

## Beyond questionnaire studies

Most studies examining the psychological impact of the COVID-19 pandemic relied on survey data and telephone interviews. These channels of data collection are vital but are also with their limitations. First, the sampling procedures, often based on convenience, may have left out vulnerable sub-populations. Second, the top-down questionnaire design process might have omitted areas of concern unbeknownst to the survey designer. This is particularly relevant in a rapidly evolving crisis. Third, the topic of the mental health impact of the pandemic has attracted a vast number of studies, creating *research fatigue* among study respondents (Patel, Webster, Greenberg, Weston, & Brooks, 2020), which would likely reduce both the validity and reliability of findings. As such, in this study, we sought to use an alternative form of data source – transcripts from an anonymous online counseling service – that not only does not add to the burden of respondents but also affords to answer relevant research questions that would be difficult to do so using self-report questionnaires.

## Current study

We sought to document and explain the changes in psychological distress among help-seekers in relation to the changes in COVID-19 severity in Hong Kong. Because the number of reported cases of COVID-19 was reported daily, we can readily track and assess the severity of the outbreak and its trajectory. We were interested in examining whether help-seekers became more distressed during the first year of COVID-19 or, as the CTS literature suggests, prolonged exposure in fact promoted adaptability. We made use of transcript data from Open Up, a 24/7 free online text-based counseling service in Hong Kong targeting those between the ages of 11–35 (Yip et al., 2020, 2021).

We used a big-data approach to answer three main research questions. First, was there an association between the severity of COVID-19 operationalized as the number of reported cases and the number of help-seekers mentioning the pandemic? Given the context from which our data were drawn (i.e. an online counseling service), we treated the mention of the pandemic as a proxy of concern and source of distress. We hypothesized that the change in the number of reported cases was associated with the change in the mention of the pandemic among help-seekers. Second, what were the psychological effects of COVID-19 severity? We operationalized psychological effects as the mentioning of keywords related to common mental disorders (CMDs) in their help-seeking. We hypothesized that the change in the number of reported cases was associated with the change in the mention of CMDs among help-seekers. Third, and also the most novel contribution of our study, how did the above associations change across the four waves of outbreaks? Were people more (or less) bothered by the pandemic in the later waves of the outbreak (e.g. Wave 4) than in the earlier waves? Considering both the sensitization and the habituation perspectives, we did not have an *a priori* prediction.

## Method

### Dataset and case identification

**Fig. 1.**
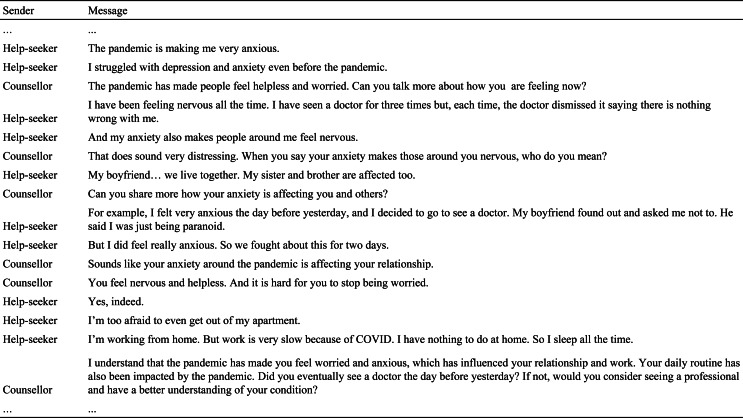
Excerpt of a fictive conversation between a help-seeker and a counsellor.

Help-seekers in Open Up communicate anonymously with professional counselors and trained volunteers via the service's web portal, SMS, WhatsApp, Facebook messenger, or WeChat. Figure 1 shows a fictive, translated excerpt of a session.

**Fig. 2.**
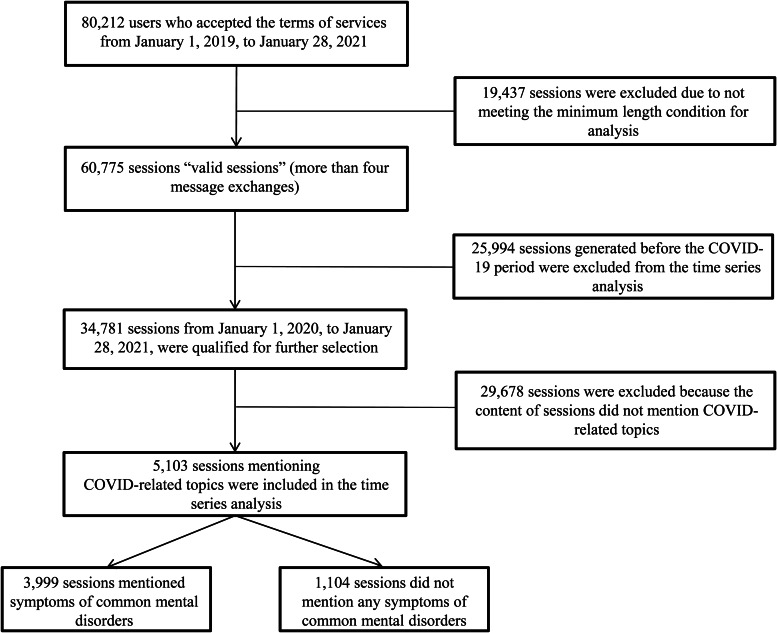
Data inclusion for COVID-19 impact analysis.

The database consisted of transcripts between January 2019 and January 2021. Among the 80 212 sessions in the database, we selected the ones with four or more message exchanges and coded them as *valid sessions*. Among the 60 775 valid sessions, 34 781 were from and after 1 January 2020, and 5103 of them mentioned COVID-19 (Fig. 2). In our analyses, we only included the texts from help-seekers; texts from the counselors were omitted.

### Different case nature

To estimate the effects of COVID-19 on help-seekers, three different types of data were analyzed. First, we extracted the daily COVID-19 reported case data in Hong Kong from the database published by the Hong Kong Government.^†^^1^ We chose this matric because the numbers of reported cases and hospitalization were very similar (10 322 and 10 291, respectively), but the former was more widely publicized. Across the study period, the total number of death was 202 (Department of Health, 2021), rendering it too low for the current analyses. Second, we coded COVID-19 mentioned sessions (‘COVID-mention’) in the dataset, which we defined as the ones in which the help-seeker mentioned any COVID-19-related terms, such as COVID, epidemic, pandemic (for the full list, see online Supplementary Table S1). Third, the sessions that mentioned COVID-19 were further broken down into those that mentioned symptoms related to a CMD (‘CMD-mention sessions’) and those that did not (‘No CMD-mention sessions’). In this study, CMDs included depressive, trauma stress, anxiety, and sleep disorders. The dictionary used (Lam, Chan, & Hamamura, 2021) was constructed based on the keywords in the diagnostic criteria in DSM 5. Substance use and eating disorders are relatively less common in Hong Kong and are not typically counted as CMDs in epidemiological studies in Hong Kong (e.g. Lam et al., 2015).^2^

### Outbreak definition

In response to the spread of the virus in China in late December 2019, the discussion of COVID-19 surged on Open Up in early January 2020, which was earlier than the report of the first confirmed case in Hong Kong on 23 January 2020. By the end of January 2021, Hong Kong has gone through four waves of COVID-19 outbreak (The Legislative Council Secretariat, 2020). While there were no official cut-off dates between waves, prior studies generally demarcate the first wave from January 2020, the second wave from March 2020, the third wave from July 2020, and the fourth wave from late November and early December of 2020 (Cheung, 2020; Chua et al., 2021). Based on the trend of reported COVID cases in Hong Kong, this study further defined Wave 1 as from 24 January to 14 February, Wave 2 as from 17 March to 11 April, Wave 3 as from 1 July to 30 August, and Wave 4 as from 20 November 2020, to 28 January 2021.

### Statistical analysis

First, we examined the trends of help-seekers who mentioned or did not mention CMDs between January 2019 and January 2021 in order to trace the changes in pattern, if any. Data were divided into three periods that demarcate two major events in Hong Kong during those two years: (1) pre-social unrest (January–May 2019); (2) the 2019 social unrest (June–December 2019); and (3) the COVID-19 pandemic (January 2020–January 2021). Our primary interest was the weekly changes in the number of help-seekers who mentioned CMDs.

Second, we evaluated the association between the number of reported COVID cases and help-seekers mentioning both CMD and COVID. The period between January 2020 and January 2021 was divided into eight epochs based on the cut-off date of four waves: pre-first-wave, first-wave, post-first-wave, second-wave, post-second-wave, third-wave, post-third-wave, and the fourth-wave. Their ordinal association with the reported COVID-19 cases was evaluated by Kendall rank correlation.

The autoregressive integrated moving average (ARIMA) model was adopted where the response time-series can be expressed as a linear combination of its own past values, past errors, and current and past values of other time-series (Benvenuto, Giovanetti, Vassallo, Angeletti, & Ciccozzi, 2020; Chakraborty & Ghosh, 2020; Hamilton, 2020). The lagged scatter plots of the time-series data were examined together with the stationarity test statistics by the augmented Dickey-Fuller (ADF) and Phillips-Perron (PP) to identify serial correlation of the data and relations to external factors. Different combinations of autoregressive (AR) and moving average (MA) orders were tested. The autocorrelation function (ACF) and the partial ACF plots helped to confirm the adequacy of the chosen model. Finally, model fit was measured by Akaike Information Criterion (AIC), Schwartz Bayesian Criterion (SBC), and Mean Square Error (MSE), where the model with the lowest AIC, SBC, and MSE was identified as the best model. The residual in the ACF plot of the fitted model and the autocorrelation test were used to ensure the appropriateness of the chosen model.

A weekly intervention model was used to measure the impact of COVID-19 on help-seekers. A daily record of COVID-19 discussion was examined for each of the four waves of the outbreak. Autoregressive components together with daily reported COVID-19 cases were explored in the daily ARIMA models. To evaluate the impact of each wave, impulse functions (i.e. the four waves) together with wave magnitudes (i.e. the values of reported cases) were introduced in the ARIMA model as exogenous variables. The full list of variables of interest is reported in online Supplementary Table S2. Non-significant variables were removed from the models.

Finally, to evaluate the changes of COVID impact and the performance of the time-series model, we trained an optimal model using the first to the third waves data (i.e. before 22 November 2020) to predict COVID-mention in the fourth wave.

## Results

### Baseline comparison

**Fig. 3.**
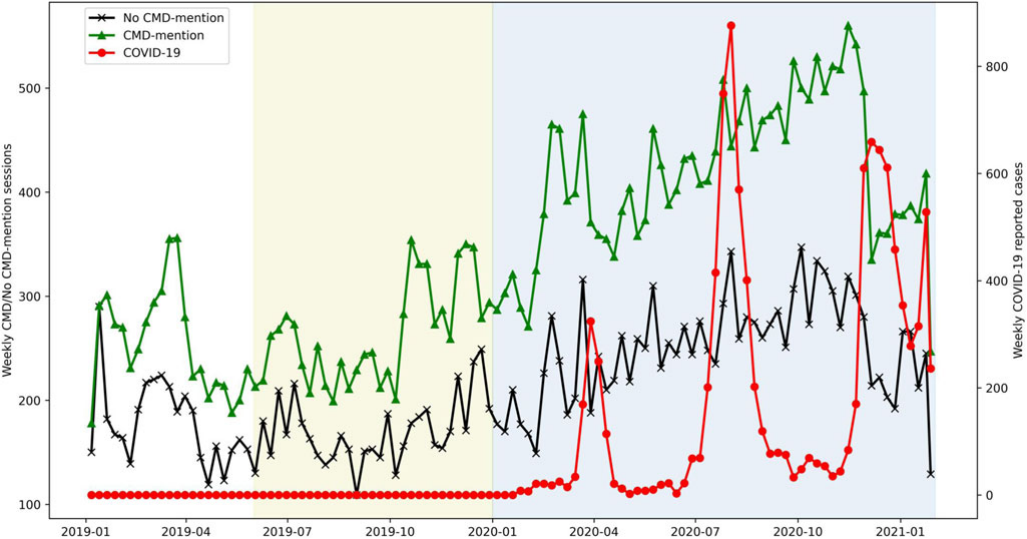
Weekly records of CMD-mention and no CMD-mention sessions across three study periods: pre-social unrest, 2019 social unrest, and COVID-19. White region demarcates pre-social unrest period, green region demarcates 2019 social unrest period, and blue region demarcates COVID-19 period.

Figure 3 presents the weekly count of valid sessions of help-seekers who mentioned or did not mention CMD between January 2019 and January 2021. A significant upward trend was observed both from those with CMD-mention (*t* = 12.7, *p* < 0.01) and no CMD-mention subgroups (*t* = 8.7, *p* < 0.01), while a remarkable drop was noted in late November 2020, which was the fourth wave of the outbreak in Hong Kong.

Table 1 reports the weekly records of help-seekers by the category of CMD mentioned. A significant increment was observed in the total valid session in the pandemic period (*p* < 0.01) and the increment varied across the CMD mentioned. Across all three study periods, symptoms of depression were the most commonly mentioned among the included CMDs, followed by traumatic stress.

**Table 1.** Weekly average records by categories of CMD mentioned across the three study periods

Period	T1: Jan 2019–May 2019 (pre-2019 social unrest)	T2: Jun 2019–Dec 2019 (2019 social unrest)	T3: Jan 2020–Jan 2021 (COVID)	T1 *v*. T2^a^	T2 *v*. T3^a^	T1 *v*. T3^a^	*F*	*p*	*η*^2^
Number of weeks	21	31	57	–	–	–	–	–	–
Valid sessions	433.9	433.3	665.9	(−68.9 to 70.0)	(−287.4 to −177.8)	(−294.8 to −169.3)	72.0	<0.001	0.58
CMD	255.3 (59%)	263.2 (61%)	417.0 (63%)	(−51.5 to 35.8)	(−188.2 to −119.3)	(−201.1 to −122.2)	83.2	<0.001	0.61
Depression	224.3 (52%)	231.7 (53%)	363.3 (55%)	(−46.6 to 31.7)	(−162.5 to −100.7)	(−174.4 to −103.7)	76.1	<0.001	0.59
Traumatic stress	90.1 (21%)	94.2 (22%)	159.8 (24%)	(−21.1 to 12.9)	(−79.0 to −52.2)	(−85.0 to −54.3)	100.8	<0.001	0.66
Anxiety	45.6 (10%)	46.8 (11%)	90.3 (14%)	(−12.5 to 10.0)	(−52.3 to −34.6)	(−54.9 to −34.5)	98.1	<0.001	0.65
Sleep disturbance	40.4 (9%)	42.0 (10%)	71.9 (11%)	(−10.9 to 7.6)	(−37.2 to −22.6)	(−39.9 to −23.2)	69.9	<0.001	0.57
No CMD	178.6 (41%)	170.1 (39%)	249.0 (37%)	(−21.4 to 38.3)	(−102.4 to −55.2)	(−97.4 to −43.4)	41.2	<0.001	0.44

a 95% confidence limits by Bonferroni (Dunn) *t* test.

**Fig. 4.**
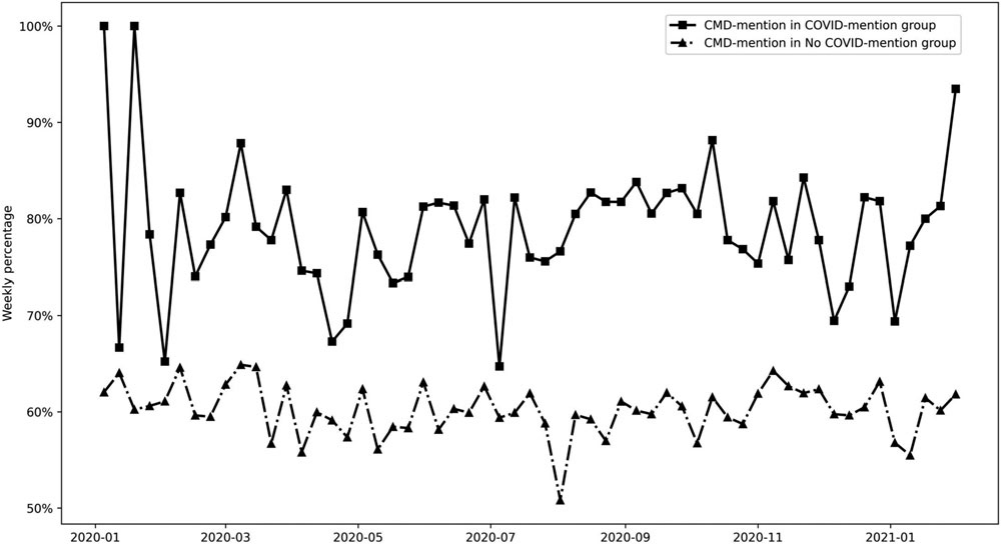
Weekly percentage of CMD-mention in COVID-mention group and No COVID-mention group.

We added a baseline comparison of CMD-mention in the COVID-mention subgroup and those who did not mention COVID (‘no COVID-mention group’). Figure 4 reports the weekly percentage of CMD-mention sessions in the two groups. The percentage of CMD-mention in the COVID-mention group (79.0%, s.d. = 6.9%) was consistently higher than the no COVID-mention group [60.2%, s.d. = 2.6%; χ^2^(1, *n* = 37 481) = 624.8, *p* < 0.001]. This further suggests the two topics were associated.

### Inter-wave comparison

Among the 5103 (14.7% of valid sessions) COVID-mention help-seekers, 3999 (78.4%) also mentioned CMDs (Table 2). A strong ordinal association between the COVID-mention and number of reported cases was observed (Kendall's *τ*-*b* = 0.642, *p* = 0.026), suggesting that the severity of the outbreak triggered more discussion among help-seekers. Furthermore, the association was mainly contributed by the CMD-mention subgroup (Kendall's *τ*-*b* = 0.642, *p* = 0.026).

**Table 2.** Weekly average of COVID-19 discussion and the reported COVID-19 case in each epoch

Weekly	Pre 1st wave	1st wave	Post 1st wave	2nd wave	Post 2nd wave	3rd wave	Post 3rd wave	4th wave
COVID-mention^a^	2.7	70	103.6	117.3	75.2	136.8	84.3	86.8
CMD^a^	2.3 (89%)	53.3 (75%)	81.8 (80%)	91.7 (78%)	56.8 (77%)	107.7 (78%)	68 (81%)	68.1 (79%)
No CMD^b^	0.3 (11%)	16.7 (25%)	21.8 (20%)	25.7 (22%)	18.3 (23%)	29.1 (22%)	16.3 (19%)	18.7 (21%)
COVID-cases	0	12	22.6	247.3	25.8	400.2	59.7	442.1

a Within the COVID-mention group, those who mentioned a common mental disorder.

b Within the COVID-mention group, no mention of a common mental disorder.

### Weekly explanatory model

#### Assumption tests

**Fig. 5.**
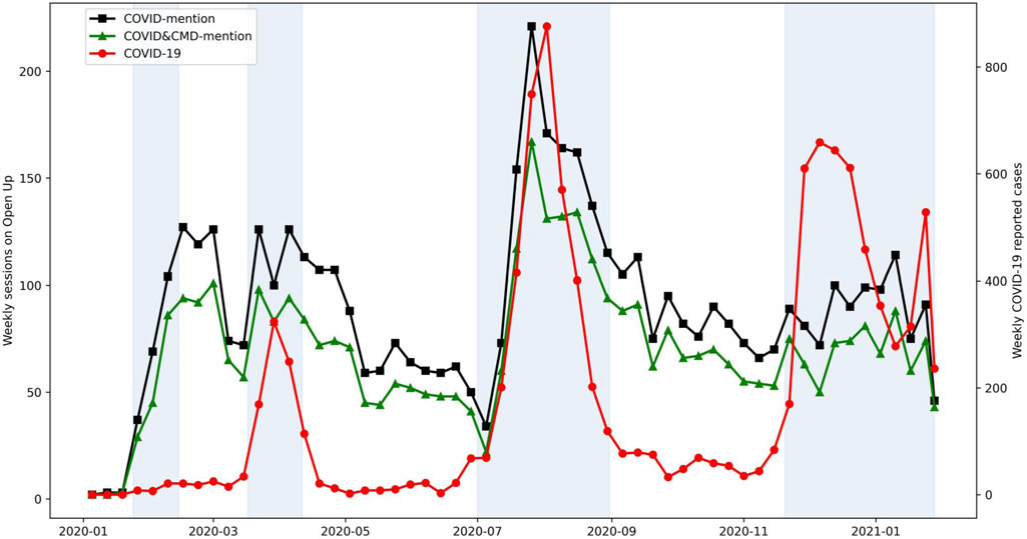
Weekly time-series of COVID-mention and COVID&CMD-mention sessions with reported COVID cases between 1 January 2020, and 28 January 2021. Blue regions demarcate the waves of COVID-19 outbreaks in Hong Kong.

Time-series data of COVID-mention suggested a stationary process with a stable variance pattern (Fig. 5). The fast decay in ACF plots, together with test statistics from ADF and PP unit root testing, confirmed the stationarity assumption. Of the models tested, the constructed ARIMA (1,0,0) model together with exogenous variables was the best fit. The number of reported cases significantly explained the data pattern of both the COVID-mention and the CMD-mention subgroup (Table 3).

**Table 3.** Parameter estimates of the selected ARIMA models for weekly COVID-mention among help-seekers in Open Up (Open Up, CMD, and No CMD)

Weekly	ARIMA model	Parameter	Estimate	Standard error	*p*	AIC	SBC
Open Up^a^	AR(1,0,0)	MU^b^	−6.34	12.77	0.620	526	532
		AR1^c^	0.77	0.09	<0.001		
		COVID^d^	0.08	0.03	0.002		
	AR(1,0,0)	MU	−4.41	9.79	0.652	520	528
		AR1	0.71	0.10	<0.001		
		ImpF-2^e^	34.87	17.14	0.042		
		MagI-3^f^	0.14	0.03	<0.001		
CMD^g^	AR(1,0,0)	MU	−4.68	10.03	0.641	497	503
		AR1	0.77	0.09	<0.001		
		COVID	0.06	0.02	0.003		
	AR(1,0,0)	MU	−3.20	7.67	0.676	492	500
		AR1	0.71	0.09	<0.001		
		ImpF-2	26.53	13.41	0.048		
		MagI-3	0.10	0.03	<0.001		
No CMD^h^	AR(1,0,0)	MU	−0.78	2.35	0.740	395	401
		AR1	0.59	0.11	<0.001		
		COVID	0.02	0.01	0.004		
	AR(1,0,0)	MU	−0.61	2.04	0.763	391	397
		AR1	0.54	0.12	<0.001		
		MagI-3	0.04	0.01	<0.001		

a Selected ARIMA model for COVID-mention.

b Intercept.

c Autoregressive, lagged by one day.

d COVID-19 reported cases in Hong Kong.

e Impulse function for the 2nd wave: the value of 1 for the 2nd wave of COVID-19.

f Magnitude effect for the 3rd wave: COVID-19 magnitude in the 3rd wave of outbreak.

g Selected ARIMA model for the CMD-mention subgroup.

h Selected ARIMA model for non-CMD mention subgroup.

#### Weekly explanatory model

The first chosen ARIMA model included a positive autoregressive component AR (1) and the number of reported cases, indicating that the frequency of a week's COVID-mention was associated with the average value of COVID-mention in the previous week and the number of reported cases in this week (Table 3). The results suggest that the significance of COVID-19 impact was mainly contributed by the impulse function from the second wave, ImpF-2: coefficient = 34.87, *p* = 0.042. The surge of reported cases in the third wave also triggered more mention of COVID-19, MagI-3: coefficient = 0.14, *p* < 0.001. A similar but milder pattern was observed in the CMD-mention subgroup. For those who did not mention CMDs, the chosen ARIMA model included a positive autoregressive component AR (1), and the significance of COVID-19 impact mainly came from the magnitude effect of the third wave.

#### Daily explanatory model

Table 4 reports the parameter estimates of the selected ARIMA models for daily COVID-mention. For the first wave, the chosen model suggests that COVID-mention was associated with number of reported cases lagged by one day, and the pattern of change was mainly contributed by the CMD-mention subgroup. The surge of cases during the second wave also triggered the growth in discussion, regardless of whether they also mentioned a CMD. In the third wave, both autoregressive component AR (1) and number of cases were significant predictors in the time-series of COVID-mention. The mention of a CMD was predicted by the previous day's discussion of CMD [AR (1)]. In the fourth wave, a simple autoregressive pattern AR (1) was observed from the CMD-mention subgroup; however, the influence of the number of reported cases was negligible.

**Table 4.** Parameter estimates of the selected ARIMA models for daily COVID-mention among help-seekers in Open Up (Open Up, CMD, and No CMD) in the four waves of COVID-19 outbreak

Daily	ARIMA model	Parameter	Estimate	Standard error	*p*	AIC	SBC
Open Up^a^	1st wave	MU^b^	0.29	1.07	0.785	129	132
		COVID^c^	0.50	0.44	0.254		
		COVID_t−1_^d^	−0.89	0.44	0.042		
	2nd wave	No model fit					
	3rd wave	MU	−0.20	1.61	0.902	399	406
		AR1^e^	0.51	0.12	<0.001		
		COVID	0.08	0.04	0.023		
	4th wave	No model fit					
CMD^f^	1st wave	MU	0.32	0.80	0.689	117	120
		COVID	0.34	0.33	0.296		
		COVID_t−1_	−0.68	0.33	0.040		
	2nd wave	No model fit					
	3rd wave	MU	−0.45	1.99	0.821	368	372
		AR1	0.70	0.09	<0.001		
	4th wave	MU	0.01	0.62	0.990	387	392
		AR1	0.27	0.12	0.021		
NO CMD^g^	1st wave	No model fit					
	2nd wave	No model fit					
	3rd wave	MU	0.00	0.30	1.000	280	284
		COVID	0.04	0.01	<0.001		
	4th wave	No model fit					

a Selected ARIMA model for COVID-mention.

b Intercept.

c COVID-19 reported cases in Hong Kong.

d COVID-19 reported cases in Hong Kong, lagged by one day.

e Autoregressive, lagged by one day.

f Selected ARIMA model for CMD- mention subgroup.

g Selected ARIMA model for non-CMD mention subgroup.

#### COVID-mention forecasting

**Fig. 6.**
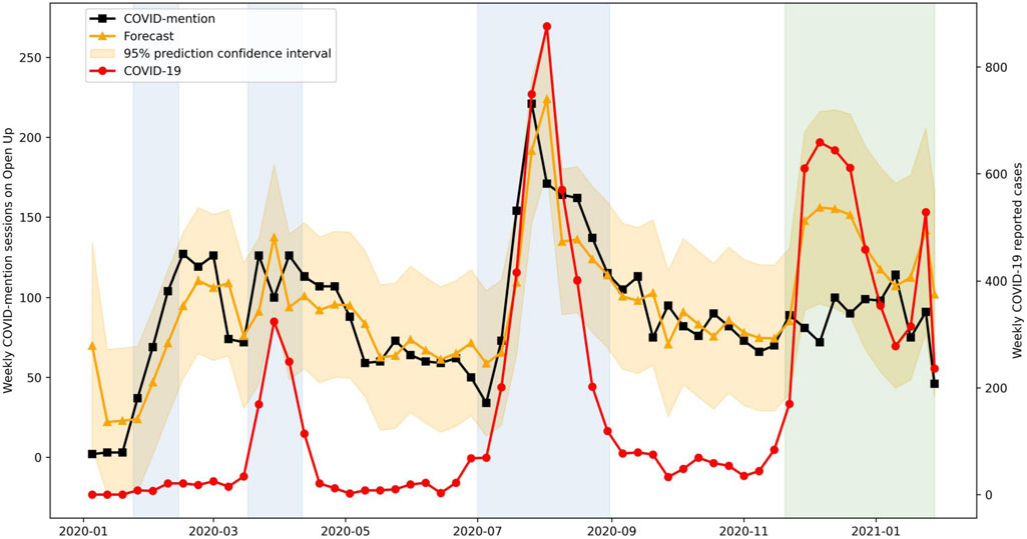
Forecast of the discussion in Open Up in the fourth wave by the chosen ARIMA model. Blue regions demarcate the first three waves of COVID-19 outbreak and green region demarcates the fourth wave.

In the aforementioned weekly model, AR (1) and weekly COVID-19 reported cases were significant predictors of COVID-mention. Using this model, we trained the first three waves of data to forecast COVID-mention in the fourth wave. The fitted lines with 95% prediction confidence interval indicate that the model adequately captured the data fluctuation of COVID-mention (Fig. 6). In forecasting the number of COVID-mention in the fourth wave, the model expected growth from 85 to 148 sessions per week in late November 2020 and a further increase to 156 sessions per week in early December 2020. The forecast overestimated COVID-mention compared with the actual data. A better forecast was achieved (within the 95% prediction intervals) after the peak of the fourth wave. These results suggest a notable decrease in COVID-mention in the fourth wave.

## Discussion

We examined the fluctuation in the psychological response across the first four waves of COVID-19 outbreak in Hong Kong. The overarching question was whether and how the psychological impact of each wave differed. Our time-series analyses of data from *Open Up*, a 24/7, free, text-based counseling service in Hong Kong, revealed several findings. First, there was a marked increase in the frequency of help-seeking in the initial months of the outbreak, compared to the prior year, which was also a turbulent one marred by the 2019 social unrest. Second, there was an association between the severity of the outbreak (in terms of reported cases) and the number of help-seekers bringing up the pandemic in session. An association was also found between the severity of the outbreak and the number of help-seekers who mentioned both the outbreak and psychological distress. Third, the said associations were not uniform across the four waves of the outbreak. The fourth wave observed a dissociation between severity and help-seekers' concern.

### Increase in number of help-seekers from 2019 to 2020

Our analysis found that there was an increase in both the traffic in Open Up and help-seekers mentioning a CMD symptom from 2019 to 2020. This suggests that there were more people seeking help for psychological problems stemming from the then little-known virus in the early months of the pandemic. This initial response is consistent with early reports on the mental health sequelae of the pandemic (McGinty et al., 2020). Hong Kong was one of the first regions to be affected by the novel disease. With the 2003 SARS epidemic, which was traumatic for many (Leung et al., 2003, 2005; Yip, Cheung, Chau, & Law, 2010), as a backdrop, the first couple of waves of the COVID-19 outbreak understandably caused distress. In this early phase, the lack of information pushed the city into a frenzy in acquiring personal protective equipment and daily essentials (Siu, 2020).

### Severity and help-seeking were associated

We found that the changes in COVID-19 discussion across the study period were explainable by the changes in actual reported cases. Similarly, the same patterns were observed in the CMD-mention subgroup; as the severity went up, so did the number of help-seeker who disclosed psychological distress. The pandemic brought upon social isolation, work and life changes, and family separation, which were associated with psychological distress under the pandemic (Elmer et al., 2020; Niedzwiedz et al., 2021; Yip & Chau, 2020). Our results confirmed that there was a non-trivial relationship between the fluctuation of outbreak severity and changes in the demand for help, especially for psychological concerns.

### Habituation effect observed in between-wave differences

The association between the severity of each wave was not consistently reflected in the concerns among help-seekers. In Wave 1, both the number of reported cases and the COVID discussions of the previous week were significantly associated with COVID discussions in the current week. Within Wave 1, the daily model suggested that COVID discussion was associated with the reported cases from the previous day. A similar pattern was also found in the CMD-mention subgroup, suggesting that those seeking help for COVID-related stressors were also those who might have mental health needs. This was reflected in both the daily and weekly models. Again, in Wave 1, perhaps because the virus was relatively novel and the amount of information about it was low, help-seekers were relatively responsive to the outbreak. As the outbreak became more severe, help-seekers also brought it up and discussed their emotional needs more with their counselors.

In Wave 2, the impulse function explained the changes in COVID-19 discussion, suggesting that the weekly discussions continued to be synchronized with the number of reported cases. However, within the second wave, none of the daily models achieved model fit, suggesting that the associations of daily discussions and COVID-19 reported cases and previous discussions were weak. One potential reason is that, in the second wave, the number of reported cases went from fewer than 10 to more than 40 a week. The rapidly deteriorating situation drove the city into a panic. Another explanation is that the time interval between the first wave and second wave was relatively short compared with the intervals between the two consecutive waves. The lasting effect of the first wave may have spilled over to the second wave.

In Wave 3, the surge of reported cases (MagI-3) triggered new peaks in the mentioning of the pandemic. Within the third wave, daily autoregressive component AR (1) and the reported cases were significant predictors of COVID discussion, suggesting that help-seekers remain vigilant and disturbed by the COVID-19 trend. The patterns were different between those who mentioned CMD and those who did not; AR (1) was a significant predictor of CMD-mention subgroup. That is, help-seekers who mentioned both COVID-19 and CMD were less affected by the changes in the number of cases than were those in the non-CMD subgroup. It is possible that some help-seekers may have habituated to the stressors and have cultivated effective coping strategies over time, as the habituation hypothesis (Stein et al., 2018) would suggest. The intervention, coping strategies, or suggestions given by the counselors may also have helped these help-seekers to adapt to the evolving situation.

In the fourth wave, only a simple autoregressive pattern AR (1) was observed from the CMD-mention subgroup. The influence of the number of reported cases was negligible. No model fit was found in this wave for the overall COVID discussion. This suggests that the changes in the number of reported cases were not reflected in help-seekers' concerns. This was also further supported by the forecasting results, where the model trained by the data from the first three waves overestimated the number of discussions in the fourth wave. This decoupling is of interest because it might reflect that the help-seekers, by the fourth wave, were less concerned by the pandemic, even though the number of cases remained relatively high. This lends support to the resilience or habituation hypothesis (Stein et al., 2018); while people were still seeking help through Open Up, their concerns and distress were less tied with the pandemic because they have either developed adaptive coping skills to mitigate the potential stress caused by the pandemic and/or they have habituated to the stressor. Whereas the sensitization hypothesis would have predicted an increase in distress with repeated exposure, our results appear to be more consistent with the habituation hypothesis, which would predict the opposite effect in the face of protracted exposure.

Our study is silent about the reasons underlying the observed habituation effect, but it does point to the possibility of adaptation. As of writing, Hong Kong has not had another wave of outbreaks while many of the neighboring countries were less fortunate. It is possible speculation that the habituation effect did not also lead to a reduction in vigilance among Hong Kong residents. The city has yet to witness an anti-mask movement, for example. Although it is still a possibility, there are no obvious reasons to interpret our results as help-seekers simply became indifferent. In other words, over time, many people – even those vulnerable enough to seek help from an anonymous counseling service – have learned to cope with the changes brought upon by the pandemic without experiencing debilitating psychological distress.

### Limitations

Our study has several limitations. First, those who use Open Up are not representative of the general public. The service is mainly for youth and young adults who are seeking psychological support; they might be relatively more vulnerable than the general public and those who seek help from other face-to-face channels (Wong, Chan et al., 2021; Wong, Wong et al., 2021). Their perception of the pandemic and its corresponding stressors might be different from those who do not, or do not need to, seek professional services. On the other hand, they might also be more proactive and savvier than those who might need help but are not resourceful enough to seek support via Open Up.

Second, our study drew inferences about help-seekers' mental health needs based on keywords related to CMD. While this dictionary contains a representative list of words related to common mental health issues, it is not exhaustive. The mention of a CMD symptom obviously does not mean the help-seeker is suffering from a disorder. Our results concerning the changes in psychological distress need to be corroborated by other forms of data.

Third, our study was conducted in Hong Kong, where the severity of the pandemic in terms of per capita mortality was relatively low. The pattern of changes in psychological distress might be very different if the casualty was a lot more severe. Our results may not be generalizable to contexts where the pandemic is acutely life-threatening.

Fourth, the availability of vaccines is a potential confounding factor explaining the dissociation between the severity of the pandemic and help-seekers' concern in the fourth wave, from 20 November 2020, to 28 January 2021. The first mass vaccination around the globe started in December 2020 (World Health Organization, 2021). The progress of vaccines might have helped relieve people's distress, which might help explain the decrease in the discussion of COVID-19 and CMDs in Open Up. However, this likelihood is attenuated by (1) the vaccination scheme in Hong Kong did not start until 22 February 2021 (Government of the Hong Kong Special Administrative Region, 2021), which was after the study period; and (2) the age groups in question were relatively skeptical about the COVID vaccines. Many Hong Kong residents, especially young people, were concerned about the safety issues caused by the haste in the development of the vaccines (Luk et al., 2021; Wong, Chan et al., 2021; Wong, Wong et al., 2021; Yan, Lai, & Lee, 2021). This skepticism was reflected in the slow vaccination uptake. The vaccination rate of people aged 12–19, 20–29, and 30–39 by May 2021 was only 2.5, 10.2, and 18.2%, respectively (Food and Health Bureau, 2021).

Lastly, we were unable to examine and control for potentially relevant demographic variables. Open Up is an anonymous platform where help-seekers use the service without the need to reveal their name, gender, age, or other demographic information. Relatedly, emerging evidence suggests that the psychological impact of the pandemic is more pronounced among disadvantaged individuals (e.g. lower income) (e.g. Kikuchi et al., 2021). Because we lacked the socioeconomic information of the help-seekers, we were unable to compare the found habituation effect across different socioeconomic classes. However, according to the Hong Kong government (Census and Statistics Department, 2021), in 2020, 93.9% of households have Internet access, 94.7% of persons aged 10–24, and 99.7% of persons aged 25–44 have at least one smartphone. Given the high penetration rate of the Internet and smartphone in Hong Kong and the free-of-charge nature of Open Up, the barrier to service for those in need was likely low.

## Conclusion

COVID-19 poses one of the greatest challenges to the health and mental health of humankind in recent history. Our study used a big data approach to analyze the transcripts of over 60 000 online text-based counseling sessions to suggest that, as the pandemic waxes and wanes, people may become habituated to its psychological toll. This can be interpreted as a form of resilience; instead of worsening with time, the psychological impact of COVID-19 may reduce over time with repeated exposure.

## Footnotes

† The notes appear after the main text.

## Notes

1 Data in Coronavirus Disease (COVID-19) DATA.GOV.HK https://data.gov.hk/en-data/dataset/hk-dh-chpsebcddr-novel-infectious-agent

2 Given the availability of data and corpus, we examined the mention of substance use disorder. Because of the relatively low mention rate (5%) and the similar trends to the ones of the included CMDs (online Supplementary Fig. S1), substance use was not included in the results reported in the main text. We were unable to examine eating disorders due to the lack of data and corpus.

## Supplementary material

For supplementary material accompanying this paper visit https://doi.org/10.1017/S0033291722000587. click here to view supplementary material

## References

[ref1] Alzueta, E., Perrin, P., Baker, F. C., Caffarra, S., Ramos-Usuga, D., Yuksel, D., & Arango-Lasprilla, J. C. (2021). How the COVID-19 pandemic has changed our lives: A study of psychological correlates across 59 countries. Journal of Clinical Psychology, 77(3), 556–570. doi: 10.1002/jclp.23082 33128795

[ref2] Bandoli, G., Campbell-Sills, L., Kessler, R. C., Heeringa, S. G., Nock, M. K., Rosellini, A. J., … Stein, M. B. (2017). Childhood adversity, adult stress, and the risk of major depression or generalized anxiety disorder in US soldiers: A test of the stress sensitization hypothesis. Psychological Medicine, 47(13), 2379–2392. doi: 10.1017/S0033291717001064 28443533 PMC5595661

[ref3] Beaglehole, B., Mulder, R. T., Frampton, C. M., Boden, J. M., Newton-Howes, G., & Bell, C. J. (2018). Psychological distress and psychiatric disorder after natural disasters: Systematic review and meta-analysis. The British Journal of Psychiatry, 213(6), 716–722. doi: 10.1192/bjp.2018.210 30301477

[ref4] Benvenuto, D., Giovanetti, M., Vassallo, L., Angeletti, S., & Ciccozzi, M. (2020). Application of the ARIMA model on the COVID-2019 epidemic dataset. Data in Brief, 29, 1–4. doi: 10.1016/j.dib.2020.105340 PMC7063124 32181302

[ref5] Bleich, A., Gelkopf, M., & Solomon, Z. (2003). Exposure to terrorism, stress-related mental health symptoms, and coping behaviors among a nationally representative sample in Israel. JAMA, 290(5), 612–620. doi: 10.1001/jama.290.5.612 12902364

[ref6] Bonanno, G., Mancini, A., Horton, J., Powell, T., LeardMann, C., Boyko, E., … Smith, T. C. (2012). Trajectories of trauma symptoms and resilience in deployed US military service members: Prospective cohort study. British Journal of Psychiatry, 200(4), 317–323. doi: 10.1192/bjp.bp.111.096552 22361018

[ref7] Bonde, J. P., Utzon-Frank, N., Bertelsen, M., Borritz, M., Eller, N., Nordentoft, M., … Rugulies, R. (2016). Risk of depressive disorder following disasters and military deployment: Systematic review with meta-analysis. British Journal of Psychiatry, 208(4), 330–336. doi: 10.1192/bjp.bp.114.157859 26892850

[ref8] Census and Statistics Department of Hong Kong Special Administrative Region. (2021). Thematic household survey report No. 73 – information technology usage and penetration. Retrieved from https://www.ogcio.gov.hk/en/about_us/facts/doc/householdreport2021_73.pdf.

[ref9] Chakraborty, T., & Ghosh, I. (2020). Real-time forecasts and risk assessment of novel coronavirus (COVID-19) cases: A data-driven analysis. Chaos, Solitons & Fractals, 135, 1–10. doi: 10.1016/j.chaos.2020.109850 PMC7190506 32355424

[ref10] Cheung, H. (2020). Covid-19: Why Hong Kong's ‘third wave’ is a warning. BBC News. Retrieved from https://www.bbc.com/news/world-asia-china-53596299.

[ref11] Chua, G. T., Wong, J. S. C., Lam, I., Ho, P. P. K., Chan, W. H., Yau, F. Y. S., … Kwan, M. Y. W. (2021). Clinical characteristics and transmission of COVID-19 in children and youths during 3 waves of outbreaks in Hong Kong. JAMA Network Open, 4(5), e218824. doi: 10.1001/jamanetworkopen.2021.8824 PMC8094012 33938934

[ref12] Courtney, D., Watson, P., Battaglia, M., Mulsant, B. H., & Szatmari, P. (2020). COVID-19 impacts on child and youth anxiety and depression: Challenges and opportunities. The Canadian Journal of Psychiatry, 65(10), 688–691. doi: 10.1177/0706743720935646 32567353 PMC7502880

[ref13] Daly, M., Sutin, A. R., & Robinson, E. (2021). Depression reported by US adults in 2017–2018 and March and April 2020. Journal of Affective Disorders, 278, 131–135. doi: 10.1016/j.jad.2020.09.065 32956962 PMC7490280

[ref14] Davies, P. T., Myers, R. L., Cummings, E. M., & Heindel, S. (1999). Adult conflict history and children's subsequent responses to conflict: An experimental test. Journal of Family Psychology, 13(4), 610–628. doi: 10.1037/0893-3200.13.4.610

[ref15] Department of Health of Hong Kong Special Administrative Region. (2021). Data in coronavirus disease (COVID-19). Retrieved from https://data.gov.hk/en-data/dataset/hk-dh-chpsebcddr-novel-infectious-agent.

[ref16] Dougall, A. L., Herberman, H. B., Delahanty, D. L., Inslicht, S. S., & Baum, A. (2000). Similarity of prior trauma exposure as a determinant of chronic stress responding to an airline disaster. Journal of Consulting and Clinical Psychology, 68(2), 290–295. doi: 10.1037/0022-006X.68.2.290 10780129

[ref17] Elliott, S., Drummond, M. J., Prichard, I., Eime, R., Drummond, C., & Mason, R. (2021). Understanding the impact of COVID-19 on youth sport in Australia and consequences for future participation and retention. BMC Public Health, 21, 1–16. doi: 10.1186/s12889-021-10505-5 33673812 PMC7935002

[ref18] Elmer, T., Mepham, K., & Stadtfeld, C. (2020). Students under lockdown: Comparisons of students’ social networks and mental health before and during the COVID-19 crisis in Switzerland. PLoS ONE, 15(7), 1–22. doi: 10.1371/journal.pone.0236337 PMC7377438 32702065

[ref19] Food and Health Bureau of Hong Kong Special Administrative Region. (2021). Daily count of vaccination by age groups. Retrieved from https://data.gov.hk/en-data/dataset/hk-fhb-fhbcovid19-vaccination-rates-over-time-by-age.

[ref20] Garfin, D. R., Holman, E. A., & Silver, R. C. (2015). Cumulative exposure to prior collective trauma and acute stress responses to the Boston Marathon bombings. Psychological Science, 26(6), 675–683. doi: 10.1177/0956797614561043 25896419

[ref21] Government of the Hong Kong Special Administrative Region. (2021). COVID-19 vaccination scheme starts. Retrieved from https://www.news.gov.hk/eng/2021/02/20210226/20210226_174848_473.html.

[ref22] Hamilton, J. D. (2020). Time series analysis. Princeton, NJ: Princeton University Press.

[ref23] Hammen, C., Henry, R., & Daley, S. E. (2000). Depression and sensitization to stressors among young women as a function of childhood adversity. Journal of Consulting and Clinical Psychology, 68(5), 782–787. doi: 10.1037/0022-006X.68.5.782 11068964

[ref24] Irish, L., Ostrowski, S. A., Fallon, W., Spoonster, E., van Dulmen, M., Sledjeski, E. M., & Delahanty, D. L. (2008). Trauma history characteristics and subsequent PTSD symptoms in motor vehicle accident victims. Journal of Traumatic Stress, 21(4), 377–384. doi: 10.1002/jts.20346 18720390

[ref25] Kikuchi, H., Machida, M., Nakamura, I., Saito, R., Odagiri, Y., Kojima, T., … Inoue, S. (2021). Development of severe psychological distress among low-income individuals during the COVID-19 pandemic: Longitudinal study. BJPsych Open, 7(2), E50. doi: 10.1192/bjo.2021.5 33583484 PMC7884661

[ref26] Labarda, C. E., & Chan, C. S. (2018). Sleep disturbances, posttraumatic stress, and psychological distress among survivors of the 2013 Super Typhoon Haiyan. Psychiatry Research, 266, 284–290. doi: 10.1016/j.psychres.2018.03.019 29609982

[ref27] Lam, C., Chan, C. S., & Hamamura, T. (2021). Time-dependent association between mass protests and psychological distress on social media: A text mining study during the 2019 anti-government social unrest in Hong Kong. Journal of Affective Disorders, 291, 177–187. doi: 10.1016/j.jad.2021.05.007 34044337

[ref28] Lam, L. C.-W., Wong, C. S.-M., Wang, M.-J., Chan, W.-C., Chen, E. Y.-H., Ng, R. M.-K., … Bebbington, P. (2015). Prevalence, psychosocial correlates and service utilization of depressive and anxiety disorders in Hong Kong: The Hong Kong Mental Morbidity Survey (HKMMS). Social Psychiatry and Psychiatric Epidemiology, 50(9), 1379–1388. doi: 10.1007/s00127-015-1014-5 25660760

[ref29] Leung, G. M., Ho, L. M., Chan, S. K., Ho, S. Y., Bacon-Shone, J., Choy, R. Y. L., … Fielding, R. (2005). Longitudinal assessment of community psychobehavioral responses during and after the 2003 outbreak of severe acute respiratory syndrome in Hong Kong. Clinical Infectious Diseases, 40(12), 1713–1720. doi: 10.1086/429923 15909256

[ref30] Leung, G. M., Lam, T. H., Ho, L. M., Ho, S., Chan, B., Wong, I. O. L., & Hedley, A. J. (2003). The impact of community psychological responses on outbreak control for severe acute respiratory syndrome in Hong Kong. Journal of Epidemiology & Community Health, 57(11), 857–863. doi: 10.1136/jech.57.11.857 14600110 PMC1732323

[ref31] Liang, L., Ren, H., Cao, R., Hu, Y., Qin, Z., Li, C., & Mei, S. (2020). The effect of COVID-19 on youth mental health. Psychiatric Quarterly, 91, 841–852. doi: 10.1007/s11126-020-09744-3 32319041 PMC7173777

[ref32] Luk, T. T., Zhao, S., Wu, Y., Wong, J. Y.-H., Wang, M. P., & Lam, T. H. (2021). Prevalence and determinants of SARS-CoV-2 vaccine hesitancy in Hong Kong: A population-based survey. Vaccine, 39(27), 3602–3607. doi: 10.1016/j.vaccine.2021.05.036 34034950 PMC8130539

[ref33] McGinty, E. E., Presskreischer, R., Han, H., & Barry, C. L. (2020). Psychological distress and loneliness reported by US adults in 2018 and April 2020. JAMA, 324(1), 93–94. doi: 10.1001/jama.2020.9740 32492088 PMC7270868

[ref34] McLaughlin, K. A., Conron, K. J., Koenen, K. C., & Gilman, S. E. (2010). Childhood adversity, adult stressful life events, and risk of past-year psychiatric disorder: A test of the stress sensitization hypothesis in a population-based sample of adults. Psychological Medicine, 40(10), 1647–1658. doi: 10.1017/S0033291709992121 20018126 PMC2891275

[ref35] Moore, S. A., Faulkner, G., Rhodes, R. E., Brussoni, M., Chulak-Bozzer, T., Ferguson, L. J., … Tremblay, M. S. (2020). Impact of the COVID-19 virus outbreak on movement and play behaviours of Canadian children and youth: A national survey. International Journal of Behavioral Nutrition and Physical Activity, 17, 1–11. doi: 10.1186/s12966-020-00987-8 32631350 PMC7336091

[ref36] Nearchou, F., Flinn, C., Niland, R., Subramaniam, S. S., & Hennessy, E. (2020). Exploring the impact of COVID-19 on mental health outcomes in children and adolescents: A systematic review. International Journal of Environmental Research and Public Health, 17, 1–19. doi: 10.3390/ijerph17228479 PMC7698263 33207689

[ref37] Niedzwiedz, C. L., Green, M. J., Benzeval, M., Campbell, D., Craig, P., Demou, E., … Katikireddi, S. V. (2021). Mental health and health behaviours before and during the initial phase of the COVID-19 lockdown: Longitudinal analyses of the UK Household Longitudinal Study. Journal of Epidemiology & Community Health, 75(3), 224–231. doi: 10.1136/jech-2020-215060 32978210 PMC7892383

[ref38] Nuttman-Shwartz, O., & Shoval-Zuckerman, Y. (2016). Continuous traumatic situations in the face of ongoing political violence: The relationship between CTS and PTSD. Trauma, Violence, & Abuse, 17(5), 562–570. doi: 10.1177/1524838015585316 25966968

[ref39] Patel, S. S., Webster, R. K., Greenberg, N., Weston, D., & Brooks, S. K. (2020). Research fatigue in COVID-19 pandemic and post-disaster research: Causes, consequences and recommendations. Disaster Prevention and Management, 29(4), 445–455. doi: 10.1108/DPM-05-2020-0164 33679011 PMC7932124

[ref40] Ronen, T., Rahav, G., & Appel, N. (2011). Adolescent stress responses to a single acute stress and to continuous external stress: Terrorist attacks. Journal of Loss and Trauma, 8(4), 261–282. doi: 10.1080/15325020305878

[ref41] Siu, J. (2020). Coronavirus: Rice, toilet paper and dried goods fly off shelves as rumours spark panic buying in Hong Kong. South China Morning Post. Retrieved from https://www.scmp.com/news/hong-kong/health-environment/article/3049237/coronavirus-rice-toilet-paper-and-dried-goods-fly.

[ref42] Smid, G. E., van der Velden, P. G., Lensvelt-Mulders, G. J., Knipscheer, J. W., Gersons, B. P., & Kleber, R. J. (2012). Stress sensitization following a disaster: A prospective study. Psychological Medicine, 42(8), 1675–1686. doi: 10.1017/S0033291711002765 22126800

[ref43] Stein, J. Y., Levin, Y., Gelkopf, M., Tangir, G., & Solomon, Z. (2018). Traumatization or habituation? A four-wave investigation of exposure to continuous traumatic stress in Israel. International Journal of Stress Management, 25(S1), 137–153. doi: 10.1037/str0000084

[ref44] Stevens, G., Eagle, G., Kaminer, D., & Higson-Smith, C. (2013). Continuous traumatic stress: Conceptual conversations in contexts of global conflict, violence and trauma. Peace and Conflict: Journal of Peace Psychology, 19(2), 75–84. doi: 10.1037/a0032484

[ref45] The Legislative Council Secretariat. (2020). Challenges and economic impacts arising from coronavirus disease 2019. Press Releases of the government of the Hong Kong Special Administrative Region. Retrieved from https://www.info.gov.hk/gia/general/202012/14/P2020121400394.htm.

[ref46] Vindegaard, N., & Benros, M. E. (2020). COVID-19 pandemic and mental health consequences: Systematic review of the current evidence. Brain, Behavior, and Immunity, 89, 531–542. doi: 10.1016/j.bbi.2020.05.048 32485289 PMC7260522

[ref47] Wilson, H. W., Berent, E., Donenberg, G. R., Emerson, E. M., Rodriguez, E. M., & Sandesara, A. (2013). Trauma history and PTSD symptoms in juvenile offenders on probation. Victims & Offenders, 8(4), 465–477. doi: 10.1080/15564886.2013.835296 PMC3834597 24273468

[ref48] Wong, K., Chan, C. S., Chan, M., Wong, C., Cheng, Q., Xiong, C., … Yip, P. (2021). Who seeks help online? Comparing online and offline help-seeking preferences among youths with suicidal ideation. Journal of Affective Disorders, 292, 21–29. doi: 10.1016/j.jad.2021.05.056 34087633

[ref49] Wong, K., Wong, E. L.-Y., Ho, K.-F., Cheung, A. W.-L., Yau, P. S.-Y., Dong, D., … Yeoh, E.-Y. (2021). Change of willingness to accept COVID-19 vaccine and reasons of vaccine hesitancy of working people at different waves of local epidemic in Hong Kong, China: Repeated cross-sectional surveys. Vaccines, 9(1), 62. doi: 10.3390/vaccines9010062 33477725 PMC7832291

[ref50] World Health Organization. (2021). Coronavirus disease (COVID-19): vaccines. Retrieved from https://www.who.int/news-room/questions-and-answers/item/coronavirus-disease-(covid-19)-vaccines.

[ref51] Yan, E., Lai, D. W. L., & Lee, V. W. P. (2021). Predictors of intention to vaccinate against COVID-19 in the general public in Hong Kong: Findings from a population-based, cross-sectional survey. Vaccines, 9(7), 696. 10.3390/vaccines9070696. 34202016 PMC8310118

[ref52] Yip, P., Chan, W. L., Cheng, Q., Chow, S., Hsu, S. M., Law Y. W., … Yeung, T. K. (2020). A 24-hour online youth emotional support: Opportunities and challenges. The Lancet Regional Health – West Pacific, 4, 1–3. doi:10.1016/j.lanwpc.2020.100047 PMC8315660 34327390

[ref53] Yip, P., & Chau, P. H. (2020). Physical distancing and emotional closeness amidst COVID-19. Crisis, 41(3), 153–155. doi: 10.1027/0227-5910/a000710 32299225

[ref54] Yip, P. S. F., Chan, W-L., Chan, C. S., He, L., Xu, Y., Chan, E., … Xu, Z. (2021) The opportunities and challenges of the first three years of Open Up, an online text-based counselling service for youth and young adults. International Journal of Environmental Research and Public Health, 18(24), 13194. 10.3390/ijerph182413194. 34948802 PMC8701729

[ref55] Yip, P. S. F., Cheung, Y. T., Chau, P. H., & Law, Y. W. (2010). The impact of epidemic outbreak: The case of severe acute respiratory syndrome (SARS) and suicide among older adults in Hong Kong. Crisis, 31(2), 86–92. doi: 10.1027/0227-5910/a000015 20418214

